# Neddylation: A Versatile Pathway Takes on Chronic Liver Diseases

**DOI:** 10.3389/fmed.2020.586881

**Published:** 2020-10-19

**Authors:** Jiping Yao, Xue Liang, Yanning Liu, Min Zheng

**Affiliations:** The State Key Laboratory for Diagnosis and Treatment of Infectious Diseases, National Clinical Research Center for Infectious Diseases, Collaborative Innovation Center for Diagnosis and Treatment of Infectious Diseases, The First Affiliated Hospital, College of Medicine, Zhejiang University, Hangzhou, China

**Keywords:** neddylation, HBV, NAFLD, liver fibrosis, HCC, therapy, MLN4924

## Abstract

Neddylation is a ubiquitin-like posttranslational modification that conjugates neural precursor cell expressed developmentally downregulated-8 (Nedd8) to specific substrates for regulation of protein activity. In light of current researches, the neddylation pathway is aberrant in the pathogenesis of many diseases. In our review, we summarize the versatile roles of neddylation in chronic liver diseases (CLDs). CLDs are one of the leading causes of chronic disease-associated deaths worldwide. There are diverse etiologic agents causing CLDs, mainly including hepatitis B virus (HBV) infection, nonalcoholic fatty liver disease (NAFLD), chronic exposure to alcohol or drugs, and autoimmune causes. So far, however, there remains a paucity of effective therapeutic approach to CLDs. In this review, we summarized the role of the neddylation pathway which runs through the chronic hepatitis B/NAFLD–liver fibrosis–cirrhosis–hepatocellular carcinoma (HCC) axis, a canonical pattern in the process of CLD development and progression. The dysregulation of neddylation may provide a better understanding of CLD pathology and even a novel therapeutic strategy. Correspondingly, inhibiting neddylation via MLN4924, a small molecule compound targeting NEDD8-activating enzyme (NAE), can potently alleviate CLD progression and improve the outcome. On this basis, profiling and characterization of the neddylation pathway can provide new insights into the CLD pathology as well as novel therapeutic strategies, independently of the etiology of CLD.

## Introduction

Chronic liver disease (CLD) is posing a significant public health problem worldwide for it causes ~2 million deaths annually. It is desperately needed to pay high attention to it (1). There are diverse etiologic agents causing CLDs, primarily including chronic infection of hepatitis B or C viruses (HBV or HCV), chronic aberrant metabolic conditions [nonalcoholic fatty liver disease (NAFLD)], chronic consumption of alcohol or drugs, and abnormal autoimmunity (2, 3). Further, the onset, development, and deterioration of CLD are progressive processes. A variety of factors are involved in them, such as inflammatory cell infiltration, liver tissue reconstruction, fibrogenesis, and extracellular matrix (ECM) deposition (4, 5). Therefore, the pathological process of CLD is complicated, characterized by pan-cellular and pan-pathway mechanisms. Recently, more and more attention has been paid to the ubiquitination pathway due to its potential role in the therapy of CLD, particularly hepatocellular carcinoma (HCC), the advanced stage of CLD.

A new insight into the CLD field is the emerging role of the neddylation pathway. Neddylation is a ubiquitination-like modification. However, unlike ubiquitination that mainly targets proteins doomed to be degraded, neddylation regulates proteins' function and stability (6). The best-known physiological substrates of neddylation are the cullin family, the crucial component of cullin-RING ligases (CRLs). CRL is the largest family of ubiquitination E3 ligases (7) and responsible for about 20% of cellular protein degradation via proteasome (8). Neddylation of cullin activates CRLs (9) and promotes the ubiquitination of substrates (10). Hence, neddylation modulates the cellular function in some degree via manipulating ubiquitination E3 ligases behind the scene. Previous researches demonstrated that neddylation inhibition can inactivate CRLs (11) and then results in accumulation of their substrates, such candidates including chromatin licensing and DNA replication factor 1 (CDT1) (12), p21 (13), and nuclear factor erythroid 2-related factor 2 (NRF2) (14, 15). Most substrates of CRLs are tumor suppressors (16). The consequence of neddylation inhibition is cell apoptosis, cell autophagy, cell senescence, and ultimately cancer suppression (17) (Table 1). Current researches of noncullin substrates of neddylation uncover that neddylation might participate in additional biological process of cells (41) (Table 2). On this basis, recent studies have uncovered that neddylation inhibition can repress HBV survival (64), alleviate steatosis (65), reduce liver fibrosis (66), and restrain pro-tumor inflammation (67). Considering the progress that has been made to understand the role of neddylation, it is worthy to explore and conclude the relevance between neddylation and CLD. In this review, we particularly focus on the role of the neddylation pathway in CLD pathology and assess the therapeutic approach targeting neddylation in CLD.

**Table 1:** Neddylation inhibition and CRL inactivation.

**Response**	**Substrate**	**CRL**	**Function**	**Reference**
Apoptosis	CDT1	CRL1^SKP^/CRL4^CDT2^	DNA damage	(12, 18, 19)
	pIkBα	CRL1^β−*Trcp*^	NF-κB inactivation	(17, 20, 21)
	NOXA	RBX2-associated CRLs	DNA damage	(22–24)
	ATF4	RBX1–CRL1	DR5 expression	(25–28)
Autophagy	DEPTOR	CRL1^β−*Trcp*^	mTORC1 inactivation	(29–31)
	HIF1α	CRL2^VHL^	mTORC1 inactivation	(32–34)
Senescence	p21	CRL1^SKP2^/CRL4^CDT2^	G2 (or G2/M) phase arrest	(13, 35–37)
	P27	CRL1^SKP2^	G2 (or G2/M) phase arrest	(38)
	WEE1	CRL1	G2 (or G2/M) phase arrest	(39)
Redox Homeostasis	NRF2	CRL1/CRL3	scavenging ROS	(14, 15, 40)

**Table 2:** Neddylation pathway substrates.

**Type**		**Substrates**	**E3 ligases**	**Function of neddylation**	**References**
Ubiquitin E3 ligases		CRLs	RBX1/2 and/or DCN1	Increases CRL activity	(42)
		Smurf	Smurf	Increases Smurf activity	(43)
		Parkin		Increases parkin activity	(44)
		VHL		Promotes VHL association with fibronectin	(45)
		BRAP2		Inhibits NF-κB-mediated transcription	(46)
		MDM2	MDM2	Increases MDM2 stability	(47)
Transcription regulation		p53	MDM2 and SCF^FBXO11^	Inhibits p53 transcriptional activity	(47)
		p73	MDM2	Inhibits p73 transcriptional activity	(48)
		IKKγ	TRIM40	Inhibits NF-κB activity	(49)
		BCA3		Inhibits NF-κB-mediated transcription	(50)
		E2F1		Reduces E2F-1 stability	(51)
		APP/AICD		Inhibits AICD-mediated transcriptional activation	(52)
		HIF1α/HIF2α		Increases protein stability	(53)
		HuR	Mdm2	Increases stability and nuclear localization	(54)
Signaling pathways	RTK signaling	EGFR	c-CBL	Facilitates EGFR degradation	(55)
		TGFβRII	c-CBL	Increases TGF-βRII stability	(56)
		SHC		Promotes Erk activation	(57)
	Apoptosis	drICE		Inhibits apoptosis	(58)
		Caspases/IAPs/RIP1		Suppresses caspase activity	(59)
	DNA damage	Histone H4	RNF111	Activates DNA damage-induced ubiquitination	(60)
	Nucleolar stress signaling	L11	MDM2	Increase stability and nucleolar	(61)
		S14		localization	(62)
	Oxidative/calcium stress	RCAN1		Increase RCAN1 stability	(63)

## Neddylation in CLD

### Overactivation of Neddylation

Deregulation of the neddylation pathway has been described in various pathological conditions. Focusing on the CLD context like liver fibrosis and HCC, both NAE1 and global protein neddylation expressions are upregulated (66, 68). However, the initial triggering mechanism that overactivates neddylation pathways during CLD is not understood exactly. Significantly, a previous study demonstrated that diverse stress conditions such as heat shock and oxidative stress may lead to an entire increase of the neddylation pathway *in vitro*. Under the stress circumstance, the ubiquitin E1 enzyme Ube1, rather than NAE1, regulates the conjugation of the NEDD8 (69, 70). Further analysis reveals that the crosstalk between NEDD8 and ubiquitin causes a prompt and dramatic amplification of the NEDD8 proteome under stress conditions. In addition, neddylation of the substrate competing with its ubiquitination upon stress could stabilize its protein level and prevent its degradation (69). Subsequently, accumulated substrates mediate vital biological processes and trigger diverse cellular responses, which may result in multiple hepatic dysfunctions eventually. Besides, in HCC, a decrease in deneddylating enzyme NEDP1 with a concomitant increase of NEDD8 conjugates leads to the inhibition of ATPase activity of heat shock protein 70 (HSP70) (71). As a result, the formation of the apoptosome is disturbed, and subsequently, the apoptosis resistance of hepatoma cells is elevated (72).

## Neddylation and HBV Infection

HBV chronic infection is a primary pathogeny leading to CLDs, especially cirrhosis and HBV-related HCC (64). HBV-encoded X protein (HBx) is a small regulatory protein that exhibits pleiotropic activities, including affecting transcription, DNA repair, cell growth, and apoptotic cell death (73). HBx can interact with various cellular proteins to influence its own activity. Among all these HBx-interacting proteins, the damage-specific DNA binding protein 1 (DDB1) is a well-characterized HBx binding partner (74). This interaction is worthy of attention because DDB1 is the adaptor protein of CRL4 ubiquitin E3 ligase. Previous researches reported that HBx–DDB1 interaction is indispensable in HBx-induced viral genome replication and affects cell viability. An interesting structural study revealed that HBx contains an α-helical motif termed the H-box, which is shared by several DDB1–CUL4-associated factor (DCAF) proteins (75). DCAF proteins act as well-known CRL4 substrate acceptors. According to the above, it is possible that HBx assembles an HBx–DDB1–CUL4–ROC1 E3 ligase complex (CRL^HBx^) as CRL4 and HBx plays the role of substrate receptor. HBx targets host proteins that suppress HBV genome replication, and CRL^HBx^ promotes their ubiquitylation and degradation via the proteasome pathway (75–77). Neddylation activates CRL4^HBx^ via conjugating Nedd8 to the cullin protein's conserved lysine residues to affect HBV replication indirectly.

**Figure 1:**
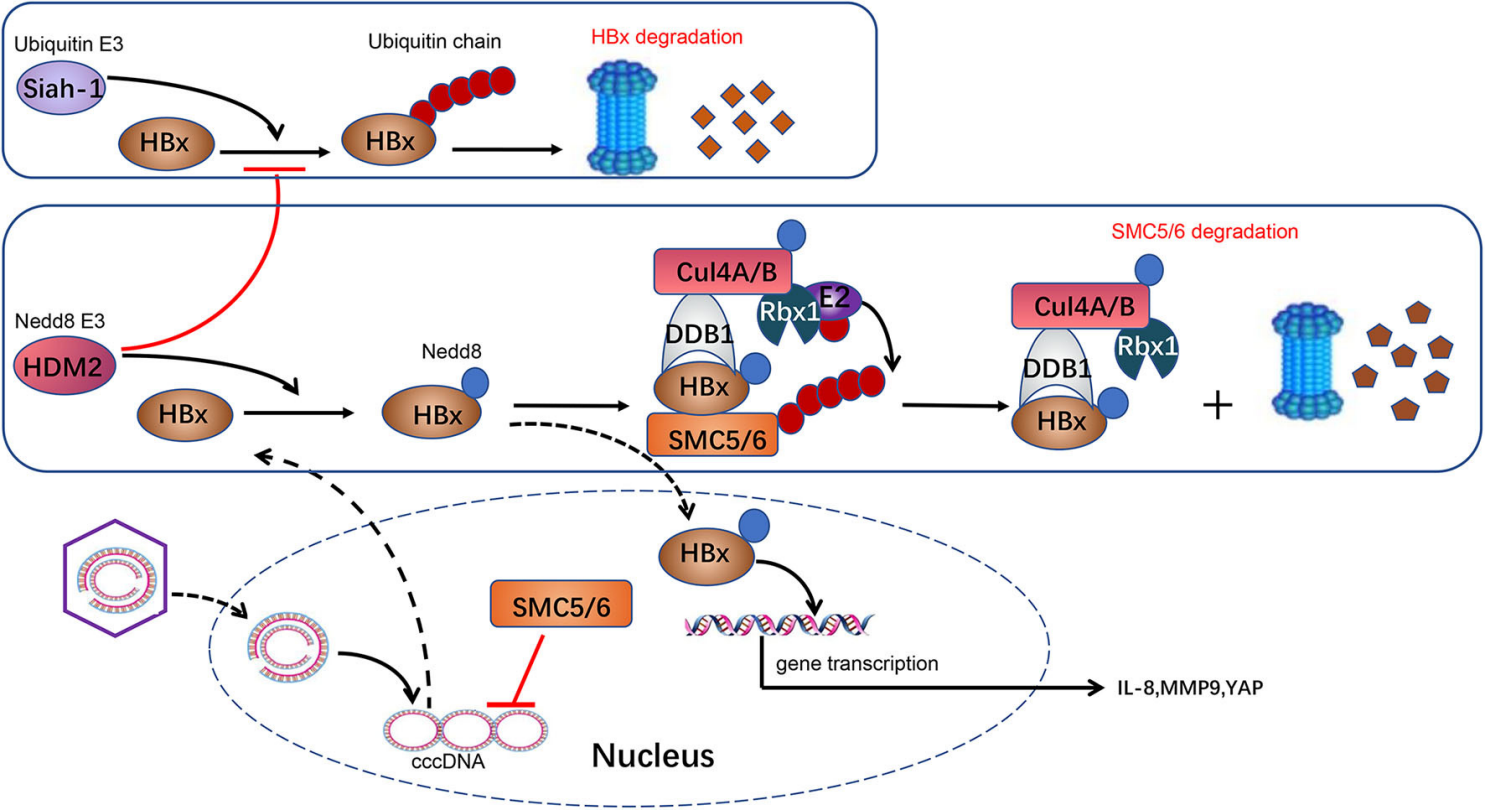
Neddylation and HBV infection. HBV is a DNA virus carrying a 3.2-kb genome. Following entry into the host cell, the 3.2-kb genome is transported into the nucleus and converted to a cccDNA, which serves as a crucial template for HBV transcription. HBx is transcripted from cccDNA and serves as a transcriptional activator to promote the expression of IL-8, MMP9, and YAP, which are implicated in HCC development. HBx in cytoplasm is ubiquitylated by E3 ligase Siah-1 to induce HBx degradation through proteasome. HBx can be neddylated by Nedd8 E3 ligase HDM2 in the cytoplasm. HDM2-mediated neddylation modification increases HBx stability by preventing its ubiquitination-induced degradation. Meanwhile, HBx plays a role as a substrate acceptor and assembles CRL4^HBx^ ubiquitin E3 ligases. HBx targets SMC5/6, a host protein that suppresses HBV genome replication, and CRL4^HBx^ promotes its ubiquitylation and degradation via proteasome. Besides, neddylation activates CRL4^HBx^ via conjugating Nedd8 to cullin protein's conserved lysine residues to affect HBV replication indirectly.

Liu et al. also found that HBx can be neddylated by the Nedd8 E3 ligase human homolog of mouse double minute 2 (HDM2). Neddylation modification alters HBx's half-life and enhances its stability. Moreover, it also increases HBx's chromatin localization and the binding with DDB1 (64). Previous researches show that HBx is ubiquitylated by E3 ligase Siah-1 to induce HBx degradation (78). Interestingly, Liu and his colleagues found that the mechanism of HDM2-mediated neddylation modification increases HBx stability by preventing its ubiquitination-induced degradation (64). These processes ultimately favor HBx's activity of transcriptional regulation, cell proliferation, and HBV-driven tumor growth. In conclusion, it provides an insight into the neddylation's role in HBV invasion. Further study found that HDM2's expression is positively correlated with HBx expression in HBV-related HCC samples (Figure 1). HDM2 has the potential to act as a new prognostic marker for HBV-related HCC. Inhibition of the neddylation pathway may provide us with a novel therapeutic method for HBV-related HCC.

## Neddylation and NAFLD

NAFLD is an increasingly prevalent CLD and has become a prominent healthy concern globally due to dietary structure change and lifestyle change (79). NAFLD is characterized by steatosis, a pathologic phenomenon of excessive triglyceride accumulation in hepatocytes. NAFLD will frequently progress to its more severe form called nonalcoholic steatohepatitis (NASH), which consists of hepatic steatosis, inflammation, and fibrosis. NASH accompanied with advanced fibrosis may eventually lead to cirrhosis and even HCC (80, 81). Recently, more and more insight has been shed on the association between NAFLD and neddylation.

Neddylation plays a crucial physiological role in lipid metabolism. Recent research reported that liver-specific deficiency of NEDD8 or UBA3 causes neonatal death with spontaneous fatty liver in mouse models. Interestingly, electron transfer flavoproteins (ETFs), whose defects can lead to fatty acid oxidation disorder in glutaric aciduria type II (GA-II), are neddylation substrates. Hepatic neddylation modification can stabilize ETFs and even enhance ETF expression via suppressing their ubiquitination, which prevents fasting-induced steatosis (82).

Furthermore, another research of serine-rich splicing factor 3 (SRSF3) illuminates that neddylation is involved in the pathology of NAFLD and NASH. It suggested that the low expression of SRSF3 is correlated with an increased risk of NAFLD, NASH, or cirrhosis. In the condition of oxidative stress, SRSF3 could be modified by the NEDD8 protein at lysine 11, which results in the degradation of SRSF3 via proteasome (83). On this basis, the result above implies that intervening with the neddylation of SRSF3 contributes to its stability and accumulation, which is beneficial for preventing hepatic steatosis, fibrosis, and inflammation.

Recently, it was reported that sterol regulatory element-binding protein 1c (SREBP1c), a critical role in maintaining lipid homeostasis, is upregulated in liver to contribute to the progress of hepatic steatosis. However, unlike SRSF3, neddylation of SREBP1c competing with its ubiquitination facilitates its stability and, rather than promoting its degradation via proteasome, eventually contributes to hepatic steatosis. Further, SREBP1c can be neddylated by NEDD8 E3 ligase HDM2, which can also mediate the neddylation of HBx as described above (84).

Additionally, Dehnad et al. revealed that advanced glycation end product (AGE) clearance receptor AGER1 was decreased in NASH. Further analysis demonstrated that exposure to high AGEs promotes an AGER1/RAGE imbalance and subsequently promotes NRF2 degradation via neddylation of cullin3, which eventually causes downregulation of AGER1 (85).

Taken together, these studies come to the conclusion that a neddylation-dependent pathway is implicated in liver steatosis and fibrosis mainly via regulating the stability of its substrates, which function as critical regulators in the process of liver steatosis. Revealing the role of neddylation in hepatic lipid metabolism and fibrosis progression may pave the way for a novel therapeutic approach in NAFLD and NASH (65).

## Neddylation and Liver Fibrosis

Liver fibrosis is a continuous wound-healing progress leading to sustained scarring response (86). Liver fibrosis can be triggered by underlying etiologies of CLDs, such as viral infection, alcohol, and NASH (87). The pathology progress is a dynamic and reversible response that can be regulated by halting or reversing the fibrosis to cirrhosis and HCC (3). To date, specific therapies validated as being effective for liver fibrosis have primarily been etiology induced. However, there is no thoroughly validated antifibrosis therapy that is independent of the underlying etiology in the clinic.

More recently, deregulated neddylation is founded in liver fibrosis (66). Firstly, neddylation inhibition decreases liver inflammation. It is reported that neddylation inhibition reduces the expression level of pro-inflammatory cytokines and chemokines, such as tumor necrosis factor α (TNFα), interleukin-6 (IL-6) and its receptor, the tumor necrosis factor alpha receptor (TNFR1) (88), the chemokine (C-X-C motif) ligand 1 (Cxcl1), the chemokine (C-X-C motif) ligand 2 (Cxcl2), the chemokine (C-C motif) ligand 2 (Ccl2), and the C-C chemokine receptors (Ccr) (Ccr1, Ccr2, and Ccr5) (89). Secondly, neddylation plays a role in hepatic stellate cell activation. In bile duct ligation (BDL) and carbon tetrachloride (CCl_4_)-induced injury rodents, neddylation inhibition decreases HSC activation and collagen accumulation, certified by reduced levels of the pro-fibrogenic factor, transforming growth factor (TGFβ), and the expression of collagen type I alpha 1 (ColIα1) (90). Thirdly, neddylation inhibition decreases fibrosis by inducing HSC apoptosis due to c-Jun accumulation. c-Jun, associated with apoptosis in several types of cells, such as HSC, could be targeted by neddylated cullins for degradation via the ubiquitin proteasome system. This provides a critical clue that neddylation inhibition could somehow modulate c-Jun levels and concomitant apoptosis (91).

Moreover, there are another two possible regulated mechanisms of neddylation in liver fibrogenesis. The transcription factor NF-κB mediates transcription of genes, such as pro-inflammation cytokines involved in the inflammation progress of fibrogenesis (92). Neddylation functions on NF-κB activation by promoting its nuclear translocation via activating SCF^β*TrCP*^'s cullin. SCF^β*TrCP*^ is a CRL that targets the NF-κB inhibitory protein IκBα for degradation via proteasome (93). It is reasonable to believe that neddylation inhibition could ameliorate inflammation and fibrosis via reducing specific NF-κB target genes' expressions. Another possible mechanism is associated with TGFβ. TGFβ is significantly important in HSC activation and involved in Smad2 signaling through transmembrane receptor serine/threonine kinases (94). An intriguing research shows that Casita B-lineage lymphoma (c-Cbl) can function as a Nedd8 E3 ligase of the type II receptor (TGFβ-RII) beyond CRL (56). The neddylation modification of TGFβ-RII interferes with its ubiquitination–proteasome degradation in blood cells. Therefore, we can speculate that the neddylation modification of TGFβ-RII stabilizes its function and thereby promotes TGFβ signaling, playing a key role in HSC activation (66, 95).

## Neddylation and HCC

HCC, the most common and frequent primary liver cancer, is the advanced stage of CLD. Although some alteration of signal molecules involved in HCC progression is discovered, the high mortality and the poor prognosis make it the third malignancy that leads to cancer-related death globally (96). Recently, it was reported that the dysregulation of the neddylation pathway is associated with HCC. The whole neddylation pathway including NEDD8; NEDD8-specific E1, E2, and E3; and even deneddylation enzymes was upregulated in HCC (97).

Inhibiting the neddylation pathway can significantly suppress HCC cell proliferation and migration, induce apoptosis, and eventually inhibit HCC growth and metastasis (98). Nieves Embade and his colleagues found that Hu antigen R (HuR) was implicated in the above process. HuR plays a critical role in hepatocyte proliferation, survival, differentiation, and HCC transformation via enhancing the stability of target mRNAs (92). The stability of HuR itself in cells is attributed to Mdm2-mediated neddylation. The neddylation modification of HuR promotes its nuclear localization and reduces its degradation (54, 99). SREBP-1 is not merely a critical regulator of lipid metabolism but is also associated with cancer metabolism. In metastatic HCC samples, SREBP-1 is upregulated with a concomitant increase of UBC12. Recently, it was reported that SREBP-1 can be neddylated by NEDD8 E3 ligase UBC12. Consequently, neddylation of SREBP-1 competing with its ubiquitination promotes the stability of SREBP-1 (68). Otherwise, liver kinase B1 (LKB1) and Akt kinases, critical regulators in proliferative metabolism of the liver, could be neddylated to enhance their stability. Inhibition of neddylation leads to metabolic reprogramming and concomitant apoptosis of liver cancer cells via altering the stability of LKB1 and Akt (100).

Importantly, neddylation is closely related to regulation of autophagy in liver cancer cells (101). Autophagy is a cellular biological process in response to various stresses to maintain cellular homeostasis. Several researches have reported that autophagy could play a pro-survival role in cancer cells. Inhibition of neddylation would induce autophagy to promote survival of cancer cell and thus result in drug resistance. Mechanistically, suppressing neddylation causes inactivation of CRL1^β−*TrCP*^ and CRL2^VHL^ and subsequently contributes to the accumulation of their substrates: DEPTOR and HIF1α. DEPTOR and the HIF1–REDD1–TSC1 axis would induce mTOR inactivation, which partially leads to autophagy (34, 102, 103). Additionally, increased level of reactive oxygen species (ROS) and activating transcription factor 3 (ATF3) could also trigger autophagy in the circumstance of neddylation inhibition (104). Recently, it was reported that inactivation of CRL4 would block polyubiquitination and proteasomal degradation of the WD repeat domain, phosphoinositide interacting 2 (WIPI2) via inhibition of neddylation, which eventually induces autophagy during mitosis (105).

**Figure 2:**
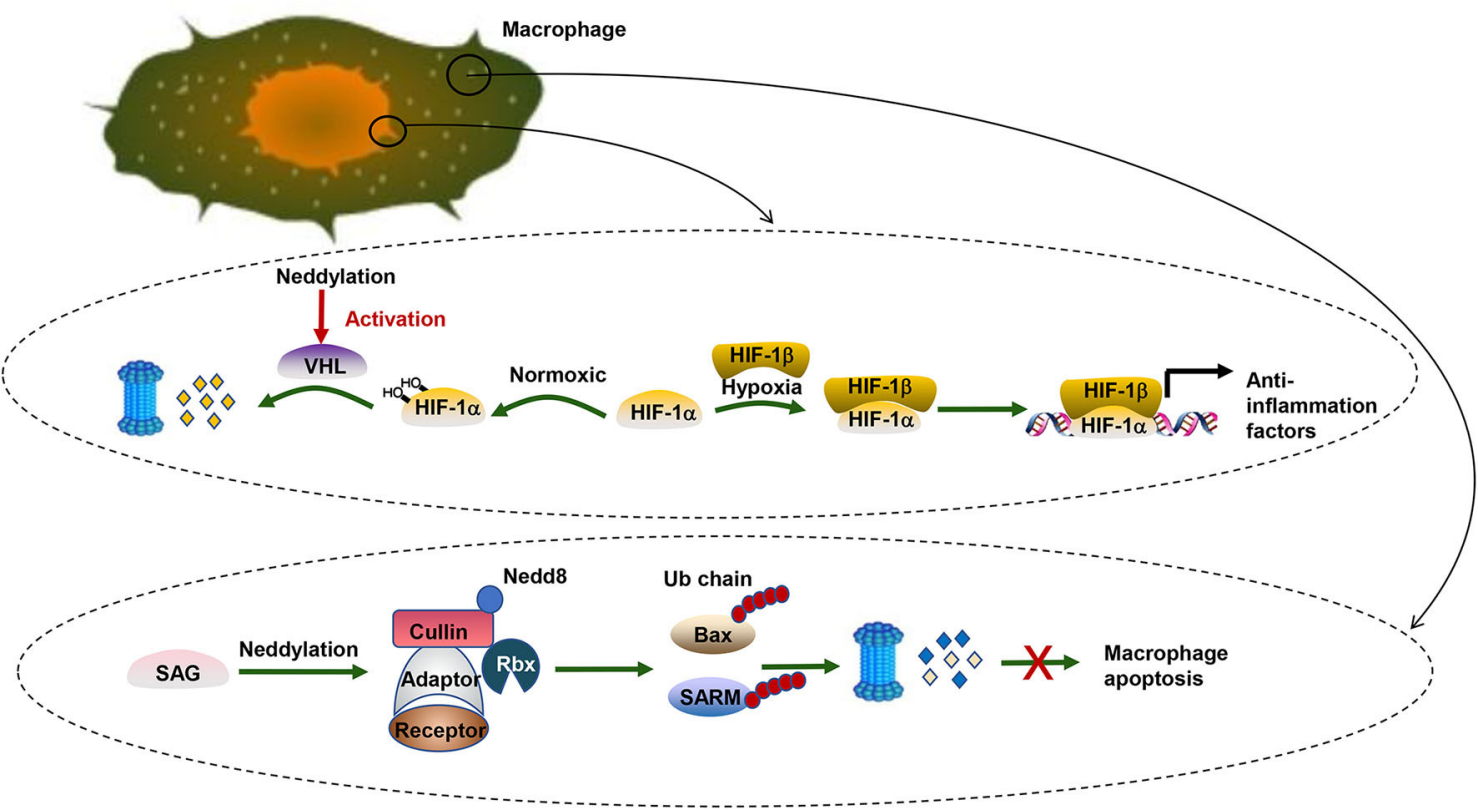
Neddylation and HCC. There are two biological processes taking place in pro-inflammation macrophages. One is related to the transcription factor HIF. HIF-1α can be targeted by pVHL, a substrate receptor of CRL2. Under normoxic conditions, HIF-1α prefers to be hydroxylated and then recognized by pVHL for degradation via the UPS pathway. Under hypoxic conditions, HIFα tends to translocate to the nucleus and form a heterodimer with a HIF1β subunit and transcriptionally regulates a wide spectrum of genes significant for the anti-inflammatory response. The other is about the apoptosis inhibition of pro-inflammation macrophages. SAG, a neddylation E3 ligase, collaborates with UPS to promote survival of infectious macrophages via ubiquitination of Bax and SARM. In contrast, SAG knockdown leads to the accumulation of proapoptotic Bax and SARM and breaks the balance between antiapoptotic Bcl-2 and Bax in the mitochondria, which induces the death of macrophages.

Moreover, neddylation also regulates pro-tumorigenic inflammation in liver cancer cells. Hypoxia-inducible factor-1 (HIF1), an oxygen homeostasis transcription factor, provides anti-inflammation activity under hypoxia conditions (106). The alpha subunit of HIF1 (HIF1α) can be targeted by the Von-Hippel-Lindau protein (pVHL), a substrate receptor of CRL2 (107). Under normoxic conditions, HIF1α prefers to be hydroxylated and then recognized by pVHL, which mediates the degradation of hydroxylated HIF1α by the UPS pathway (32, 33). Under hypoxic conditions, HIF1α tends to translocate to the nucleus and form a heterodimer with a HIF1β subunit and transcriptionally regulates a wide spectrum of genes significant for the anti-inflammatory response (108). Likewise, neddylation modification of cullin2 alters the activity of CRL2 and eventually influences the stability of HIF1α (109) (Figure 2). Recently, Cannito et al. (110) have suggested that SerpinB3, a serine protease inhibitor, can stimulate proliferation of hepatic tumor cells and subsequently facilitate HCC progression by enhancing the stabilization of HIF2α by promoting the direct and selective neddylation of HIF2α (111).

Further analysis elucidates that the role of neddylation poses a profound effect not only on liver cancer cells but also on immune cells. In macrophages, blocking neddylation regulates NF-κB signaling and eventually causes the downregulation of proinflammatory cytokines. Besides, further research reveals that the sensitive to apoptosis gene (SAG), a neddylation E3 ligase, collaborates with UPS to promote survival of infectious macrophages via degrading proapoptotic Bax and sterile α and HEAT/armadillo-motif-containing protein (SARM) (112). SAG also affects cytokine secretion of macrophages (113). Similar to innate immune cells, SAG-deficient T cells also show decreased proliferation, reduced production of cytokines, and diminished release of the T-cell lineage. Besides, knockdown of Ubc12 in CD4+ T cells caused impaired T-cell receptor/CD28-induced proliferation because T cells were arrested in the G0/G1 phase of the cell cycle (113). Moreover, cytokine production like IL-2 and the differentiation of CD4+ T cells into effector Th-cell subsets are decreased when the expression of Ubc12 is reduced. The neddylation pathway regulates various aspects of CD4+ T-cell function. However, the exact mechanism remains to be investigated. A similar result was found in B cells (114–116).

## Therapeutic Strategies Targeting Neddylation

MLN4924 (pevonedistat), a small molecule inhibitor of NAE, disrupts CRL-mediated protein turnover; causes restraining tumor cell growth by inducing apoptosis, senescence, and autophagy; and causes sensitization to chemoradiation therapies in a cellular context-dependent manner (8). Currently, MLN4924 is being evaluated in several phase Ib/II/III clinical trials (https://www.clinicaltrials.gov/). Among these clinical trials, five completed phase I clinical trials in solid tumors and hematological malignancies have verified that MLN4924 is safe and feasible. Several phase II clinical trials are under way. Particularly, a phase III clinical trial of MLN4924 combined with azacytidine is currently recruiting volunteers with hematological malignancies. Previous researches suggested that MLN4924 is a potent and selective inhibitor in hematological neoplasms as well as many solid neoplasms, including HCC. Given that the summaries about previous studies of MLN4924 in HCC are relatively sufficient, we primarily demonstrate the use of MLN4924 in HBV infectious, NAFLD, and liver fibrosis.

Currently, although nucleoside or nucleoside analogs can suppress new viral DNA replication, they are unable to eradicate the cccDNA from infected hepatocytes thoroughly (117). This is the most critical reason resulting in HBV rebound and obstinacy. Recently, it is reported that MLN4924 can suppress HBV transcription and protein expression significantly via restoring the structural maintenance of chromosome (SMC) complex protein (SMC5/6) levels (118). Murphy et al. (119) identified that SMC5/6, a host restriction that restricts HBV replication by inhibiting HBV gene expression, is the substrate of CRL^HBx^ (120). HBx targets SMC5/6 for degradation by the ubiquitin–proteasome system, and subsequently, the transcription from cccDNA is elevated (Figure 1). Neddylation of cullin is necessary for activation of CRL^HBx^. MLN4924 inhibits the neddylation process, promotes SMC5/6 accumulation, and ultimately leads to restriction of viral transcription and HBV DNA level, particularly cccDNA (118). Thereby, the neddylation pathway is a potential target for HBV treatment. MLN4924 may become a novel anti-HBV agent, though evidence is far from being enough.

In view of the role of neddylation in regulating stabilization of SRSF3, MLN4924 can repress the degradation of SRSF3 and reduce the accumulation of SREBP1c, which alleviate the steatosis and prevent the progression of NAFLD (65). Serrano-Macia et al. have found that β-oxidation activity and ketone body levels were enhanced after treatment. On the contrary, the levels of lipid peroxidation and ROS are significantly reduced after MLN4924 treatment. Further study suggests that in a NASH mouse model, MLN4924 treatment can reverse steatosis, inflammation, and fibrosis. Hence, inhibition of neddylation via MLN4924 is a potent therapeutic option because it can ameliorate fatty acid metabolism (121).

Similarly, using the pharmacological inhibitor MLN4924 could protect liver from injury, inflammation, and fibrosis via regulating the function of hepatocytes. To be more specific, MLN4924 treatment reduces the expression of pro-inflammatory cytokines previously associated with liver damage and, therefore, ameliorate the inflammation after liver injury (122). In agreement, pro-fibrogenic factors implicated in liver fibrosis, such as TGFβ, COL1α1, matrix metalloproteinase-9 (MMP9), and interferon-α (IFNα), are consistently decreased after using MLN4924. More importantly, MLN4924 diminishes the activation of HSC (66). Together, these results highlight that MLN4924 treatment is pointed out as a potential antifibrosis therapy that is independent of the underlying etiology in the clinic.

In this review, we conclude that inhibition of neddylation pharmacologically via MLN4924 can significantly alleviate CLD exacerbation and progression in view of recent researches (8). Given its well-tolerated toxicity and potent antitumor activity in preclinical trails, MLN4924 is anticipated to be a promising therapeutic approach for CLD. However, there still remains some challenges for final application of MLN4924 in the clinic. Due to the treatment-emergent NAEb mutations, resistance to MLN4924 during therapy will appear, which subsequently reduces the effectiveness of this medicine (123). Therefore, it is indispensable to design next-generation NAE inhibitors that can overcome treatment-emergent resistance.

## Conclusions and Perspectives

Chronic hepatitis/NAFLD–liver fibrosis–cirrhosis–HCC axis is a canonical pattern in the process of CLD progression (124). Recent studies in the neddylation pathway provides us with crucial clues that neddylation is a versatile pathway that takes on various aspects and phases of CLD (Table 3). Although the complicated mechanism underlying the overactivation of the neddylation pathway during CLD still remains elusive, there are several notable features of neddylation in CLD. First, the NEDD8 and NAE1 expressions are upregulated significantly in NAFLD, liver fibrosis, and HCC, and the components of the neddylation pathway like Nedd8, E1 (NAE), E2, and E3 may become novel biomarkers for CLD diagnosis. Second, under stress conditions, neddylation modification directly or by activating CRL indirectly promotes the stability of the substrate (promotes degradation in rare cases), and the accumulated substrate functions as a crucial molecule to facilitate the development of CLD. Third, MLN4924 could inhibit the overactivation of neddylation during CLD and thus alleviates the pathological process.

**Table 3:** Neddylation in CLDs.

**CLD**	**Substrate**	**CRL/E3 ligase**	**Function**	**Reference**
Hepatitis B	SMC5/6	CRL4^HBx^/HDM2	Promotes HBV replication	(77, 78)
NAFLD/NASH	ETFs	?	Promotes hepatic steatosis	(82)
	SRSF	?		(83)
	SREBP1c	HDM2		(84)
	NRF2	CRL3		(85)
Liver fibrosis	c-Jun	CRL?	Promotes activation of HSC and inflammation	(91)
	IkBα	CRL1^β−*Trcp*^		(93)
	TGFb-RII	c-CBL		(56)
HCC	HuR	Mdm2	Promotes proliferation, survival and metastasis of cancer cells	(54, 99)
	SREBP1	?	Regulates pro-tumorigenic	(68)
	LKB1	?	inflammation Regulates the function of immune cells	(100)
	Akt	?		(100)
	HIF1a	CRL2^VHL^		(106–109)
	HIF2a	?		(110)
	Bax/SARM	SAG		(112)

However, there remain several issues that need to be further explained correspondingly. First, the underlying mechanism by which the neddylation pathway is overactivated in CLD has not been demonstrated clearly so far. Second, besides HSC and cancer cells, whether and how the upregulated neddylation pathway plays a significant role in hepatic parenchyma, Kupffer cells, and tumor microenvironment require further exploration. Third, MLN4924 suppresses the whole neddylation pathway and may cause potential unforeseeable secondary effects. Moreover, MLN4924 would induce autophagy that acts as a pro-survival signal in cancer cells. Given this situation, it is probably sensible to be cautious about using MLN4924 for CLD therapy.

Notably, sumoylation, another important ubiquitin-like posttranslational modification, is identified as a double-edged sword in CLD while neddylation generally causes pathological consequences. Inhibition of the global sumoylation pathway might not always be an optimal therapeutic strategy due to its “two faces” in CLD (125–128). Therefore, targeting the neddylation pathway holds promise for the therapy of CLD. On the context of drug discovery, it is highly anticipated that more specific and safer small molecule inhibitors aiming at special targets such as specific E3 Nedd8 ligases or deneddylase enzymes should be discovered as novel therapeutic approaches for CLD.

## Author Contributions

JY wrote the manuscript and prepared figures: MZ, YL, and XL provided expert comments and edits. All authors contributed to the article and approved the submitted version.

## Conflict of Interest

The authors declare that the research was conducted in the absence of any commercial or financial relationships that could be construed as a potential conflict of interest.

## Abbreviations

CLD: chronic liver diseases
MDM2: murine double minute-2
NEDD8: neural precursor cell expressed developmentally downregulated-8
HBV: hepatitis B virus
HCV: hepatitis C virus
HCC: hepatocellular carcinoma
HBx: HBV-encoded X protein
HSC: hepatic stellate cell
NAE: NEDD8-activating enzyme
E2F-1: E2F transcription factor 1
ECM: extracellular matrix
CRL: cullin-RING ligases
VHL: von Hippel-Lindau
RBX: RING box protein-1
SKP: S-phase kinase-associated protein
CDT1: chromatin licensing and DNA replication factor
CDT2: chromatin licensing and DNA replication factor 2
NRF2: nuclear factor erythroid 2-related factor 2
NF-κB: the nuclear factor kappa-light-chain-enhancer of activated B cells
ATF4: activating transcription factor 4
βTrCP: beta-transducin repeat containing protein
DCN1: defective in cullin neddylation 1
ROS: reactive oxygen species
mTORC: mammalian target of rapamycin complex
NASH: nonalcoholic steatohepatitis
NAFLD: nonalcoholic fatty liver disease
TRIM40: tripartite motif containing 40
BCA3: breast cancer-associated protein 3
FBXO11: F-box protein 11
HuR: Hu antigen R
TGFβ-RII: transforming growth factor β type II receptor
AICD: APP intracellular domain
EGFR: epidermal growth factor receptor
BRAP2: BRCA1-associated protein 2
SCF, Skp1, cullin: and F-box protein
RTK: receptor tyrosine kinase
cccDNA: covalently closed circular DNA
IL-8: interleukin-8
MMP9: matrix metalloproteinase-9
DEPTOR: DEP domain containing mTOR-interacting protein
HIFα: hypoxia-inducible factor-α
DCAF: DDB1–CUL4-associated factor
IFNα: interferon-α
ColIα1: collagen type I alpha 1
TGFβ: transforming growth factor
TNFα: tumor necrosis factor α
IL-6: interleukin-6
Cxcl: the chemokine (C-X-C motif) ligand
Ccl: the chemokine (C-C motif) ligand
Ccr: the C-C chemokine receptors
c-Cbl: Casita B-lineage lymphoma
pVHL: Von-Hippel-Lindau protein
DDB1: the damage-specific DNA binding protein 1
SARM: sterile α and HEAT/armadillo-motif-containing protein
Bax: Bcl-2 associated protein X
CUL: cullin
KC: Kupffer cells
CCl4: carbon tetrachloride
JNK: c-Jun N-terminal kinase
Bcl-2: β-cell lymphoma 2
SMC: the structural maintenance of chromosomes
ETFs: electron transfer flavoproteins
SRSF3: serine-rich splicing factor 3
BDL: bile duct ligation
CCl4: carbon tetrachloride
HSP70: heat shock protein 70
SREBP1c: sterol regulatory element-binding protein 1c
HDM2: human homolog of mouse double minute 2
LKB1: liver kinase B1
AGEs: advanced glycation end products
WIPI2, WD repeat domain: phosphoinositide interacting 2.
